# Dietary interactions with the bacterial sensing machinery in the intestine: the plant polyphenol case

**DOI:** 10.3389/fgene.2014.00064

**Published:** 2014-04-04

**Authors:** Noha Ahmed Nasef, Sunali Mehta, Lynnette R. Ferguson

**Affiliations:** Department of Nutrition, Faculty of Medical and Health Sciences, University of AucklandAuckland, New Zealand

**Keywords:** microbiota, autophagy, phytochemicals, Toll-like receptors, Nod-like receptors

## Abstract

There are millions of microbes that live in the human gut. These are important in digestion as well as defense. The host immune system needs to be able to distinguish between the harmless bacteria and pathogens. The initial interaction between bacteria and the host happen through the pattern recognition receptors (PRRs). As these receptors are in direct contact with the external environment, this makes them important candidates for regulation by dietary components and therefore potential targets for therapy. In this review, we introduce some of the main PRRs including a cellular process known as autophagy, and how they function. Additionally we review dietary phytochemicals from plants which are believed to be beneficial for humans. The purpose of this review was to give a better understanding of how these components work in order to create better awareness on how they could be explored in the future.

## INTRODUCTION

The human body is inhabited by complex communities of microorganisms known as the microbiota which inhabit most surfaces (Hooper et al., 2012). It is estimated that there is up to 100 trillion (10^14^) bacteria (Savage, 1977; Ley et al., 2006; De Cruz et al., 2012) which is around 10-fold greater than the number of human cells in the same individual (De Cruz et al., 2012). The majority of the microbiota (10–100 trillion) inhabits the human gastrointestinal tract where they are most dense in the distal intestine (≥10^12^/cm^3^ intestinal contents). In the distal intestine the microbiota has many beneficial functions such as fermentation of indigestible dietary residues, production of vitamin K, control of intestinal epithelial cell (IEC) proliferation and differentiation, and the creation of a protective barrier against pathogens (Hooper et al., 2012).

In the intestine, a balance is required between the digestion of nutrients by symbiotic bacteria and protection against pathogenic bacteria (Hooper et al., 2012). In healthy individuals, the intestinal immune system has evolved to distinguish between normal gut microbiota and pathogenic bacteria and responds appropriately to each (Hooper et al., 2012). During bacterial infection, inflammation is activated as a defense mechanism and is generally beneficial, However, if inflammation is uncontrolled, this can lead to chronic inflammation causing disease such as inflammatory bowel disease (Medzhitov, 2010). The appropriate response to the resident microbiota begins with the microbiota sensing receptors which activates downstream signals that respond by either defense, attach, repair, or protection.

Studies have uncovered a mechanism that feeds into the bacterial sensing pathway, known as autophagy (see Autophagy). Autophagy is a mechanism used by cells for degradation of cytoplasmic material and is required for quality control and immune regulation.

Current literature suggests that dietary components can interact with processes in the host and has the potential to modify its course. One of the best studied and largest group of dietary components are phytochemicals. These compounds have a wide range of effects that include anti-inflammatory, anti-cancer, anti-oxidant, and other beneficial properties both *in vivo* and *in vitro*. However, the results are controversial and sometimes unclear. This is likely due to the differences in methods used to assess polyphenols. These findings suggest that dietary interventions have the potential to modify and prime different physiological process including immunity. Understanding these pathways further and how diet can interact with them will therefore contribute to developing personalized nutrition to manage disease.

This review will focus on introducing the bacterial sensing machinery and autophagy, and how they work. The second half of this review will introduce plant polyphenols their digestion, metabolism, bioavailability, and how they interact with the host cells to carry out their role.

## BACTERIAL SENSING

**FIGURE 1:**
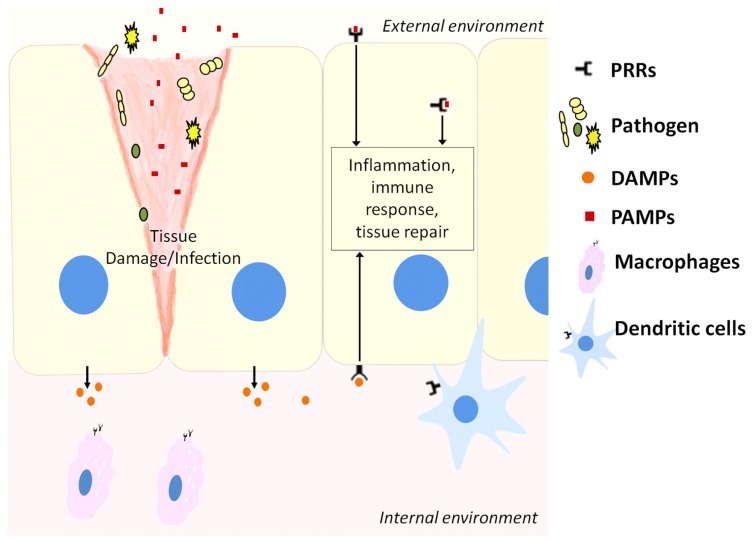
**Host cells including epithelial cells, macrophages, and dendritic cells contain pattern recognition receptors (PRRs) that can recognize molecules released from pathogens during infection (PAMPs) or stressed cells during tissue damage (DAMPs).** This activates downstream pathways to resolve infection or repair damaged tissue.

The innate immune system recognizes molecular structures that are characteristics of microbial pathogens but not mammalian cells (Abbas et al., 2012). The microbial substances that stimulate innate immunity are called pathogen-associated molecular patterns (PAMPs). Different classes of microbes express different PAMPs. The innate immunity also recognizes endogenous molecules that are produced by and released from damaged cells. These substances are known as damage-associated molecular patterns (DAMPs). Receptors that recognize PAMPs and DAMPs are expressed on phagocytes that include macrophages, neutrophils, dendritic cells (DCs), and epithelial cells that compose the barrier interface between the body and the external environment (**Figure 1**). These receptors are known as pattern recognition receptors (PRRs). When molecules bind to PRRs, they activate signal transduction events that promote the anti-microbial and pro-inflammatory functions of the cells in which they are expressed (Abbas et al., 2012).

There are several families of PRRs that have been identified (Abbas et al., 2012). These include the Toll-like receptors (TLRs), Nod-like receptors (NLRs), RIG-like receptors, and other cell-associated PRRs (Abbas et al., 2012). The most widely studied PRRs are the TLRs and NLRs which will be discussed in detail in this section.

### Toll-LIKE RECEPTORS

Toll-like receptors are a family of transmembrane proteins that recognize and respond to: (1) PAMPs (Ozinsky et al., 2000), (2) DAMPs (Piccinini and Midwood, 2010), and (3) pathogenic and non-pathogenic microorganisms in the form of microorganism-associated molecular patterns (MAMPs; Ramos et al., 2004). To date, a total of 10 TLRs have been identified in humans (TLR1–10; Abreu, 2010).

Toll-like receptors are predominantly expressed on host innate immune cells including epithelial cells (Abreu, 2010). The TLRs are comprised of type I transmembrane glycoproteins expressed on the cell surface (TLR1, TLR2, TLR4–6, and TLR10) or endosomal compartments (TLR3, TLR7–9). All TLRs express the N-terminal ectodomain that contains leucine rich repeats (LRRs) involved in ligand recognition and co-receptor interaction. They also express a transmembrane region and an intracellular region containing a Toll/IL1R resistance (TIR) signaling domain (Singletary and Milner, 2008).

Each TLR recognizes specific molecules that activate it. TLR4 is the main receptor for lipopolysaccharides (LPSs) from Gram-negative bacteria. TLR4 can also sense mannan which is the fusion (F) protein of respiratory syncytial virus (RSV) and Chlamydial heat shock protein (hsp). TLR2 interacts with TLR1 or TLR6 to recognize tri- or diacylated lipoproteins from Gram-positive bacteria, mycobacteria, or mycoplasma (Lien et al., 1999). TLR5 detects bacterial flagellin (Gewirtz et al., 2001; Mizel and Snipes, 2002). Endosomal TLRs sense viral double stranded RNA (TLR3; Alexopoulou et al., 2001), single strand RNA (TLR7 and TLR8; Diebold et al., 2004; Heil et al., 2004), and hypomethylated CpG motifs present in bacterial, viral, and fungal DNA (TLR9; Beutler, 2008).

Most TLRs form homodimers upon ligand binding. In contrast, TLR2 forms heterodimers with either TLR1 (TLR1/2) or TLR6 (TLR2/6) to respond to tri- and diacylated lipoproteins, respectively (Schenk et al., 2009). Gram-positive bacteria and mycobacteria express diacylated lipoproteins, whereas lipoproteins of Gram-negative bacteria have an additional acyl group. This puts TLR2 in a unique position of being capable of responding to lipoproteins from wide range of bacteria making it a vital bacterial sensing cell surface receptor against infection (Schenk et al., 2009).

Toll-like receptor response to molecules can be divided into two distinct intracellular pathways (Smoak et al., 2010): one leading to the activation of the MyD88-dependent pathway and the other through the TIR-domain-containing adapter-inducing interferon (IFN)-β (TRIF) signaling arm (Covert et al., 2005). All TLRs (with the exception of TLR3) use the MyD88 signaling pathway which associates with the TLRs through the TIR–TIR domain interactions (Beutler, 2008). This is followed by the recruitment of IL1R-associated kinase 4 and kinase 1 which signals downstream to activate nuclear factor kappa beta (NFκβ), mitogen-activated protein kinases (MAPKs), and inflammatory cytokines (Doyle and O’Neill, 2006). TLR3 solely engages with TRIF to activate inflammatory cytokines and type I IFNs. On the other hand, TLR4 which uses both MyD88- and TRIF-dependent pathway, also uses TRIF to signal expression of co-stimulatory molecules and type 1 IFNs via the activation of TANK-binding kinase 1 (TBK1) and IFN regulatory factor (IRF) 3 and 7 (Doyle and O’Neill, 2006; Beutler, 2008). TLR4 and TLR2 require the bridging adaptor TIR domain-containing adapter protein (TIRAP) to recruit MyD88 to the TLRs. However, TLR4 also requires a bridging adaptor TRIF-related adapter molecule (TRAM) that recruits TRIF to the TLR4 complex (Yamamoto et al., 2003). TLR4 which recognizes Gram-negative bacteria, mainly LPS, represents the principal pathway responsible for detecting and responding to endotoxins, resulting in the triggering of both the MyD88-dependent and -independent pathways (Fukata et al., 2009). Sensing of conserved PAMPs such as LPS via the LRR-containing ectodomain leads to TLR dimerization. This brings their TIR signaling domain closer to each other which form an intracellular docking platform that enables recruitment of adaptor proteins and kinases (Beutler, 2008). In one study, delayed activation of NFκβ in MyD88-deficient mouse embryo fibroblast (MEF) model cells as compared to the wild type cells was reported, suggesting that although both pathways activate NFκβ and inflammation, the TRIF-dependent pathway can do this with delayed kinetics (Covert et al., 2005). The study found that LPS-stimulated MyD88-deficient cells, in comparison with cells containing MyD88- and TRIF-deficient cells showed substantially slower kinetics to reach the initial peak for NFκβ activation. They also found the NFκβ activation in MEF cells with TRIF began much earlier after stimulation with LPS than in MEF cells deficient in TRIF. The MEF cells with and without MyD88 sustained the activation of the NFκβ levels for much longer than TRIF-deficient MEFs. These observations suggest that, while the dependent pathway triggers the response to the initial stimuli, the independent pathway is responsible for the sustenance of the pro-inflammatory program (Biswas and Tergaonkar, 2007).

In one study, TLR2 was also shown to activate a second pathway in parallel to MyD88-dependant pathway (Cario et al., 2007). The authors found that TLR2 signaling involves the PI3K–Akt pathway which modulated intestinal epithelial barrier function *in vitro* in IECs and *ex vivo* in mice. Stimulating TLR2 with the synthetic triacylated lipopeptide analog Pam3CSK4 in IEC, resulted in MyD88-dependant phosphorylation of the Akt p70S6K S6 ribosomal pathway through the PI3K pathway. In contrast stimulation with LPS did not lead to phosphorylation of Akt and its downstream substrates above baseline IEC. In their study they also found that TLR2 functions through the PI3K–Akt to attenuate the MAPK–NFκβ-signaling cascade. Overexpression of Akt leads to the significant dampening of PAM3CSK4-induced NFκβ activation *in vitro*. Their findings suggest that the PI3K–Akt secondary pathway ensures tolerance toward ligands from commensal bacteria (Cario et al., 2007). This study looked at PAM3CSK4 which activates TLR2/TLR1. Whether the same results apply to the activation of TLR2/TLR6 remains to be investigated.

### Nod-LIKE RECEPTORS

When pathogens enter the cytosol of cells they are detected by cytosolic receptors known as NLRs which elicit the appropriate response to clear or control the infection (Correa et al., 2012). They do so by recruiting a number of molecules to form a complex multi-protein structure referred to as the inflammasome (or a signalosome in the case of NOD1 and NOD2; Correa et al., 2012).

#### Structure of the NLRs and intracellular signaling via the NLRs

All NLR proteins contain: a C-terminal region characterized by a series of LRR domains that are involved in recognizing microbial components or ligands; a central nucleotide domain termed the NACHT domain that is important for self-oligomerization; and a N-terminal effector domain that is responsible for the interaction of the NLR with downstream signaling molecules (Correa et al., 2012). NLRs can vary in the number of LRRs as well as their N-terminal interacting domain. Many NLRs have been identified that include NOD1, NOD2, NLRP1, NLRP3, NLRP6, NLRP7, NLRC4, NAIP5, AIM2, and RIG-I (Chen et al., 2009).

Based on NLR’s N-terminal protein–protein interacting module, the NLRs can be divided into three subgroups depending on the interacting domain they have (Correa et al., 2012): (1) caspase recruitment domains (CARDs), (2) pyrin domains (PYDs), or (3) other domains such as baculovirus IAP (inhibitor of apoptosis) repeat domains (BIRs). The type of interacting domain that an NLR possess will determine the type of multiprotein complex recruited and the type of response achieved.

When the NOD receptors are activated by their ligands, the receptors oligomerize through mediation via the NACHT domains (Correa et al., 2012). This recruits RIP2 (receptor interacting protein 2) domain where the CARD of NOD1 or NOD2 bind the CARD of RIP2. This results in the ubiquitination by IAPs and recruitment of the linear ubiquitin chain assembly complex (LUBAC) by the X-linked inhibitor of apoptosis protein (XIAP) with further binding of TAB/TAK1 complex. TAK is an upstream activator of the IκB kinase (IKK) complex as well as the stress kinase cascades that results in JNK and p38 MAPK activation (Correa et al., 2012). In addition, NOD1 and NOD2 have been reported to interact with other NLRs that are important for caspase-1 activation (Correa et al., 2012). Most NLRs (with the exception of NOD1 and NOD2) will recruit caspase-1 either directly or indirectly (Correa et al., 2012). Caspase-1 processes a number of cellular substrates which includes the conversion of pro-IL1β and pro-IL18 into their active forms (Martinon et al., 2009). In addition to pro-inflammatory effect, continuous activation of caspase-1 can result in a form of cell death known as pyroptosis. This has characteristics of both apoptosis and necrosis (Martinon et al., 2009). NOD2 was shown to specifically and directly interact with NLRP1, NLRP3, and NLRP12, whereas NOD1 interacts only with NLRP3 (Moreira and Zamboni, 2012).

#### NOD1 and NOD2

NOD1 and NOD2 are the first NLRs that were identified and are examples of NLRs containing a CARD (Inohara et al., 1999; Ogura et al., 2001). NOD1 has been widely expressed in many cell types and tissues *in vivo*, whereas NOD2 has been found in macrophages, DCs, paneth cells, keratinocytes, intestinal epithelium, lung oral cavity, and osteoblasts. Both proteins are activated by bacterial peptidoglycan (PG; Schleifer and Kandler, 1972). PG is responsible for providing shape and mechanical rigidity to bacteria (Schleifer and Kandler, 1972). It is a major component of Gram-positive bacterial cell wall, while in Gram-negative bacteria it is found as a thin layer in the periplasmic space (Correa et al., 2012). NOD2 is a general bacterial sensor that detects and directly binds muramyl dipeptide (MDP) a motif that is present in the PGs of both Gram-positive and -negative bacteria (Girardin et al., 2003b). In contrast NOD1 is dependent on the presence of L-Ala-y-D-Glu-diaminopimelic acid (m-DAP), an amino acid characteristic of most Gram-negative and some Gram-positive bacteria (Girardin et al., 2003a). Several groups have reported a role of NOD1 in the detection of a variety of invasive Gram-negative bacteria such as *E. coli* (Kim et al., 2004) and *Chlamydia* (Opitz et al., 2005). Because PG from both Gram-positive and -negative bacteria contains MDP, NOD2 functions as a general sensor of most bacteria (Correa et al., 2012). However PG from Gram-positive bacteria do not contain m-DAP (with a few exceptions), NOD1 mainly senses products from Gram-negative bacteria (Correa et al., 2012). Moreover, several studies have demonstrated the activation of other NLRs including NLRP3 and NLRP1 by MDP (Moreira and Zamboni, 2012). The activation of these NLRs with MDP leads to the secretion of ILβ (Martinon et al., 2007).

## AUTOPHAGY

**FIGURE 2:**
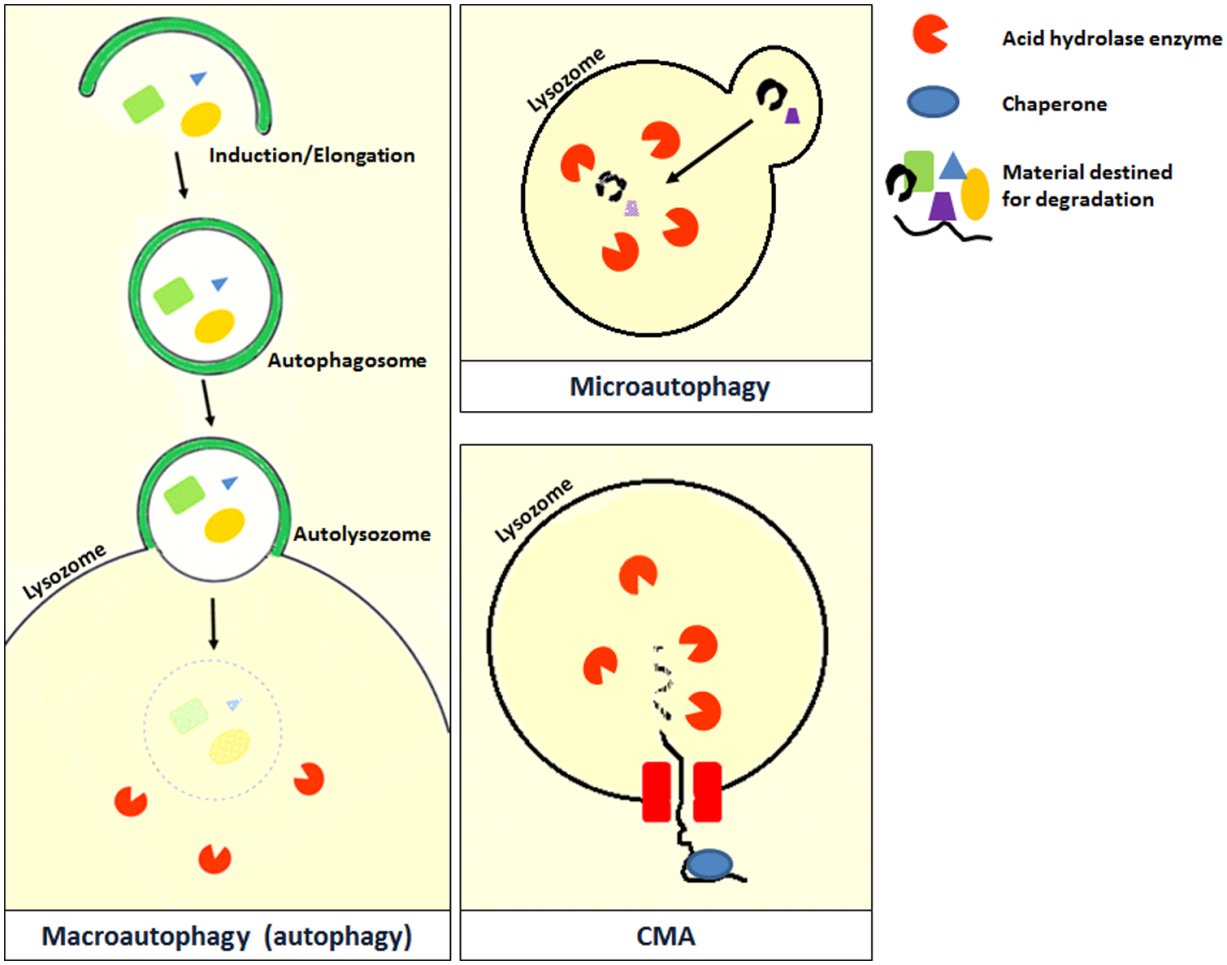
**Microautophagy occurs when bulk cytosolic components are directly engulfed by lysosomes through the invaginations of the lysosomal membrane where they are rapidly degraded by hydrolase enzymes.** This process has been studied in yeast and is still poorly characterized in mammals (Cuervo and Macian, 2012). Chaperone-mediated autophagy (CMA) is a very complex and specific pathway which is initiated when a chaperone recognizes a targeting motif in the cytosolic protein to be degraded. The chaperone/substrate complex reaches the lysosome and the substrate is internalized through the translocation complex in the lysosomal membrane (Cuervo and Macian, 2012). CMA is considerably different from the other types of autophagy because it does not directly engulf the protein material but selectively transfers it individually into the lysosome (Kadian and Garg, 2012). The most extensively described type of autophagy in the literature is macroautophagy which is also referred to as autophagy in the literature. A review on macroautophagy (which will be referred to as autophagy from this point) is provided in this review.

Autophagy is derived from the Greek word for “self-eating,” and refers to the process by which the cells breakdown and reuse their own constituents (Levine et al., 2011). Unlike proteasomes that are also involved in cellular degradation, autophagy is a recycling pathway and plays an important role in maintaining cellular homeostasis (Singletary and Milner, 2008). Autophagy can be broadly divided into three types based on the method of transfer used to deliver the cellular content into the lysosome (Cuervo and Macian, 2012). The three types of autophagy are macroautophagy, microautophagy, and chaperone-mediated autophagy (**Figure 2**; Cuervo and Macian, 2012). However, the different types of autophagy do not function in isolation but often function in an interconnected manner.

Autophagy is the main pathway that is activated in response to a number of stressors with a pro-survival function (Cuervo and Macian, 2012). In addition, some level of the basal autophagy exists in almost all cell types and contributes to maintenance of cellular homeostasis (Deretic, 2011). Autophagy either degrades or recycles the cytoplasmic content initially by the formation of an autophagosome. Autophagosomes are intermediate membrane-surrounded structures that perform two major functions: firstly it isolates the targeted cytoplasmic content within a cell from the remaining cellular matter; secondly it delivers the isolated cytoplasmic content into mammalian lysosomes or plant and yeast vacuoles. There are different types of selective autophagy that have been described according to the substrate they target (Lapaquette et al., 2012). Aggrephagy refers to degradation of aggregated proteins (Rubinsztein, 2006), pexophagy to peroxisome degradation (Iwata et al., 2006), mitophagy to mitochondria degradation (Okamoto et al., 2009), reticulophagy to ER degradation (Bernales et al., 2006), and xenophagy to the degradation of intracellular microorganisms (Deretic, 2010).

### MECHANISM OF AUTOPHAGY

Autophagy can be induced by a variety of immune signals and stress stimuli, including inflammatory cytokines, starvation and energy stress, ER stress, PAMPs and DAMPs, hypoxia, redox stress, and mitochondrial damage (Kroemer et al., 2010). Steps involved in the process of autophagy after initiation are summarized as follows:

Upon initiation, autophagy formation goes through several steps (Kroemer et al., 2010). Beclin 1 (Atg6 in yeast), UVRAG (Vps38 in yeast), Vps34 (Class III PI3K), and Vps15 are assembled to form the lipid kinase signaling complex to mediate nucleation or vesicle formation. The molecules to be digested are surrounded by the isolating membrane called the phagophore which starts to elongate (Kroemer et al., 2010). There are two ubiquitin-like conjugation systems which are part of the membrane elongation process (Kroemer et al., 2010). The first system involves the covalent conjugation of Atg12 to Atg5 with the help of Atg7. This results in the association of Atg16L1, forming the Atg16L1–Atg12–Atg5 complex. This complex functions by recruiting the lipidated form of the microtubule-associated protein 1 light chain 3 (LC3-II/Atg8 in yeast; Yang and Klionsky, 2010). Small fractions of the cytosolic ATG12–ATG5–ATG16L1 complex associate with the outer membrane of the phagophore and dissociate from it on or near completion of the double-membrane autophagosome (Mizushima et al., 2003). Atg5 and Atg16L1 depend on each other for their membrane targeting; whereas Atg12 is dispensable for Atg5–Atg16L1 membrane association (Mizushima et al., 2003).

The second system that is important in the elongation process involves the actual lipidation of LC3. This is done by the conjugation of phosphatidylethanolamine (PE) to the glycine residue of the mammalian LC3 by the sequential action of Atg4, Atg7, and Atg3 (Yang and Klionsky, 2010). LC3 is initially synthesized as its unprocessed form proLC3. LC3 is cleaved at its C-terminus by the cystine protease Atg4 into the mature form LC3-I. LC3-I is conjugated to PE by the ubiquitin E1-like protein – Atg7, and the ubiquitin E2-like protein – Atg3, to generate a smaller lipidated form of LC3, LC3-II. This lipid conjugation results in the conversion of the soluble form of LC3 (known as LC3-I) to its membrane form LC3-II. LC3-II is stably associated with the autophagosome membrane. Atg16L1 determines the site of LC3 attachment through an interaction with Golgi-resident small GTPase Rab33 (Itoh et al., 2008). LC3-II is found both on the luminal and cytosolic surfaces of autophagosomes. Elongation is then followed by the closure of the autophagosome and fusion with the lysosomal compartment and the hydrolysis of the molecules within the autophagosome (Tanida, 2011).

## DIETARY PHYTOCHEMICALS

Plant secondary metabolites also known as phytochemicals are derived from the products of primary metabolism in plants. They are defined as bioactive non-nutrient plant compounds found in fruits, vegetables, grains, and other plant foods (Liu, 2012). It is estimated that there have been more than 5,000 individual phytochemicals identified so far. However, a large percentage remains undiscovered (Shahidi and Naczk, 1995). According to the literature, phytochemicals have been classified broadly into phenolic compounds, terpenoids, nitrogen-containing compounds, alkaloids and sulfur-containing compounds, phytosterols, and carotenoids (**Table 1**; Rein et al., 2013).

**Table 1:** The different classes of phytochemicals.

Phytochemical class	Description	Reference
Phenolic compounds	At least one aromatic ring; one or more hydroxyl groups attached; more than 8000 structures; includes flavonoids and phenolic acids	Tsao (2010)
Terpenoids	Sometimes called isoprenoids; derived from five carbon isoprene units; more than 40,000 molecules; contributes to the aroma and flavor of plants	Aharoni et al. (2005)
Nitrogen-containing alkaloids	Low molecular weight, nitrogen-containing compounds; mostly derived from amino acids; found in ~20% of plant species; exploited as pharmaceuticals, stimulants, narcotics, and poisons	Wink (1998)
Sulfur-containing compounds	Glucosinolates in cruciferous crops (e.g., Broccoli); Alliins in *Allium* crops (e.g., Garlic); compartmentalized enzyme–substrate systems that produce a variety of products when the plant tissue is damaged	Mithen (2008)
Phytosterols	Plant steroids equivalent to cholesterol in animals; found mainly in vegetable oil	Ling and Jones (1995)
Carotenoids	Widely spread in plants; provides the colors yellow, red, and orange to plants; 600 known species; all contain eight isoprenes molecules; 40 carbon atoms	Kadian and Garg (2012)

Phytochemicals have a wide range of molecules, starting from the low-molecular weight phenolic acids to the highly polymerized proanthocyanidins. In addition to their broad classification, phytochemicals have been divided into two distinct classes: water soluble and lipid soluble (Neilson and Ferruzzi, 2012).

Unlike vitamins and minerals, these phytochemicals are not recognized as essential dietary components because lacking in them does not cause any specific deficiency. However, these bioactive compounds have been linked to biological activity in mammalian systems that may impact health and disease risk (Liu, 2012). Most dietary phytochemicals are considered non-essential nutrients. Therefore they are defined as compounds that can be found in the organism, but not made by the organism and not expected to be present in the organism, or used for normal metabolic function (also known as xenobiotics; Neilson and Ferruzzi, 2012). Many foods contain hundreds or even thousands of phytochemicals with variable and mostly unknown biological activity (Neilson and Ferruzzi, 2012). Of these, the polyphenols and the carotenoids are the best understood.

Phytochemicals vary widely in their composition in fruits and vegetables, nuts, and grains. This variation depends on several factors including soil (Price et al., 1989; Mansfield et al., 1999), climatic conditions (Albert et al., 2009), agricultural methods (Binns et al., 2002), physiological stress under which plants are grown (Gershenzon, 1984), degree of ripeness (Faurobert et al., 2007), storage conditions and length of storage before consumption (Liu, 2012). Thus no single value is representative of the amount of phytochemicals found in an individual plant species. The mechanism of action of different phytochemicals are often complimentary to one another and are likely to work synergistically (Liu, 2012).

Like other xenobiotics, phytochemicals are subjected to the body’s detoxification system (Neilson and Ferruzzi, 2012). This system is designed to reduce the toxicity of potentially toxic compounds. Biotransformation of xenobiotics is catalyzed by enzymes known as drug-metabolizing enzymes to enable their metabolism, detoxification and excretion from the body (Yang et al., 2010). The drug-metabolizing enzymes can be broadly classified into three groups where phase I and phase II are enzymes while phase III are transporters.

### PHASE I METABOLISM

Metabolism usually begins with the hydrolysis of polymeric, glycosylated and/esterified native compounds via the brush border of the small intestine, and the microbial enzymes (phase I metabolism; Spencer et al., 1999). Phytochemicals are usually present in food as glycosides or other conjugates and need to be hydrolyzed in order to be absorbed (Yang et al., 2010). Phase I metabolism encompasses both redox and hydrolytic reactions. The oxidation of xenobiotics in the intestine is mainly performed by a diverse family of enzymes referred to as cytochrome p450 or CYPs (Yang et al., 2010).

### PHASE II METABOLISM

Once absorbed into the IECs called enterocytes, xenobiotics are subjected to phase II metabolism by the process of conjugation (Yang et al., 2010; Neilson and Ferruzzi, 2012). Conjugation is a common detoxification reaction which reduces the number of reactive hydroxyl groups on the compound and includes glucuronidation, sulfation, methylation, acetylation, glutathione, and amino acid conjugation (Jancova et al., 2010). The process of conjugation makes the xenobiotics more polar and hydrophilic resulting in increased solubility which is necessary for urinary excretion (Dutton, 1978). This involves conjugation reactions where a hydroxyl group on a compound is modified by the addition of sulfate, glucoronic acid, or methyl group (Neilson and Ferruzzi, 2012).

Phase II drug metabolizing enzymes are mostly belonging to a group of enzymes known as transferases that catalyze the transfer of functional groups. These include UDP-glucuronosyltransferases (UGTs), sulfotransferases (SULTs), *N*-acetyltransferases (NATs), glutathione *S*-transferases (GSTs), and various methyltransferases [mainly thiopurine *S*-methyl transferase (TPMT) and catechol *O*-methyl transferase (COMT); Jancova et al., 2010]. UGT isoforms have a broad tissue distribution with a major location in the liver and the small intestine (Strassburg et al., 2000; Izukawa et al., 2009). The SULT family have been identified as either cytosolic or membrane bound and exhibit a wide distribution including the liver, brain, breast, intestine, jejunum, lung, adrenal glands, endometrium, placenta, kidney, and blood platelets (Riches et al., 2009). NATs are cytosolic enzymes found in many tissues (Windmill et al., 2000). Soluble GST is widely distributed around the body and has been found in the liver, kidney, brain, pancreas, testis, heart, lung, small intestine, skeletal muscles, prostate, and spleen (Whalen and Boyer, 1998). TPMT is a cytosolic enzyme and mainly found in the liver and kidney with low levels in the brain and lungs (Pacifici et al., 1991). COMT is an intracellular enzyme and is either a cytoplasmic soluble form or a membrane bound form located in the cytosolic side of the rough endoplasmic reticulum (Jeffery and Roth, 1984). COMT is expressed in most tissues with the highest expression in the liver, kidney, intestine, and brain (Nissinen et al., 1988; Boudíková et al., 1990; Hong et al., 1998).

The metabolites after phase II metabolism appear to be efficiently effluxed by the efflux transporters in phase III metabolism (Neilson and Ferruzzi, 2012).

### PHASE III METABOLISM

In phase III metabolism, the metabolites are either effluxed back into the intestinal lumen or to the bloodstream from where it is taken to the liver. Enterocytes are intestinal cells which act as the first barrier against xenobiotics. These cells use the action of efflux transporters to prevent the buildup of xenobiotic compounds in their cytoplasm. The transporters either returns the compounds (either in its native form or metabolized form) back into the lumen or transport them to the portal vein where they enter the liver for further processing (Neilson and Ferruzzi, 2012).

The efflux of phytochemicals is mediated by a number of transporters. The efflux of polyphenols has been shown to be facilitated by the ATP-binding cassette (ABC) superfamily of transmembrane transporters, which is termed phase III metabolism (Wakabayashi et al., 2006; Planas et al., 2012). Multidrug resistant protein (MRP) 2 is an apical/luminal end transporter that was shown to efflux the compounds back to the lumen (Lambert et al., 2007). MRP1 is a basolateral/blood stream end transporter that transport the compounds to the blood circulation (Lambert et al., 2007).

### BIOAVAILABILITY OF DIGESTED COMPOUNDS

The rate and extent to which the active compound is absorbed from its ingested form and becomes available at the site of action is defined as bioavailability (Neilson and Ferruzzi, 2012). This definition takes into account two factors *in vivo*: (1) the active compound must be present at the site of action to produce its biological activity and (2) the concentration of the compound at the site (Neilson and Ferruzzi, 2012). This definition is more easily applicable to pharmaceuticals than to dietary phytochemicals originating from complex food matrices. Bioavailability of dietary phytochemicals is not only complicated by the biochemical properties of the molecule but also because of enzyme and microbial-mediated metabolism and active efflux (Neilson and Ferruzzi, 2012).

### INTESTINAL EPITHELIAL CELLS

Intestinal epithelial cells have many functions apart from absorption. They are involved in the metabolism of food substances and also intestinal immunity. One interesting characteristic unique to IEC is that they are usually exposed to high concentrations of nutrients, non-nutrients, microbes, and xenobiotics. This suggests that the IEC’s function is affected or even regulated by external substances including food components despite their function being generally controlled by internal factors such as hormones and cytokines (Shimizu, 2010).

Several studies indicate that the small intestine has poor absorption of dietary polyphenols (Manach et al., 2005). Therefore most of the ingested dose passes through the small intestine and reaches the colon. The colon is home to a complex bacterial community that has the ability to extensively ferment unabsorbed material (van Duynhoven et al., 2011; Neilson and Ferruzzi, 2012).

### PLANT POLYPHENOLS

Polyphenols are a large structurally diverse group of organic compounds that contain at least one aromatic ring with one or more hydroxyl groups attached (Rein et al., 2013). Plant foods contain many different types of polyphenols, which are increasingly seen as effective protective agents against disease (Scalbert et al., 2002, 2005; Shapiro et al., 2007; Priego et al., 2008; Bravo, 2009; Del Rio et al., 2010; Sies, 2010; Gonzalez et al., 2011). Polyphenols represent a wide range of compounds which are divided into several classes determined by their structure. These include phenolic acids, flavonoids, still beans, and ligands (Del Rio et al., 2010). In this section, plant polyphenols will be reviewed in detail.

#### Absorption of polyphenols

Polyphenols ingested from food remain outside the body until they are absorbed through epithelial cells lining the gastrointestinal tract (Neilson and Ferruzzi, 2012). Most intact polyphenol absorption happens in the small intestine with further absorption occurring in the colon of the large intestine (Scalbert et al., 2002). In order to be absorbed by the epithelial cells in the gut several factors need to take place. First, polyphenols must be released from any interactions with other food components (Neilson and Ferruzzi, 2012). This is done by mechanical action such as chewing and grinding in the mouth. Further breakdown happens in the stomach via the gastric juices (Neilson and Ferruzzi, 2012). Second, the stability of the polyphenols in the intestine will greatly impact the concentration reaching the epithelial surface. Third polyphenols must be soluble in the bulk aqueous phase of the gastrointestinal milieu in order to facilitate diffusion through the unstirred water layer that protects the epithelial surface layer (Neilson et al., 2009).

According to Lipinski’s Rule of 5, compounds that have five or more hydrogen bond donors (OH and NH groups), 10 or more hydrogen bond acceptors (notably N and O), a molecular weight of greater than 500, and a log *P* greater than 5 are usually poorly absorbed after oral administration. This is because of their large actual size (high molecular weight), high polarity or large apparent size (due to the formation of a large hydration shell; Yang et al., 2008). Dietary polyphenols range from species that violate the Lipinski’s rule and as such have been shown to have poor bioavailability (Lipinski et al., 2001; Mulder et al., 2001; Yang et al., 2008), while others have been shown to have good absorptive characteristics as predicted by the Lipinski’s rules (Yang et al., 2008).

It is believed that the absorptions of polyphenols into the epithelial cells of the small intestine (enterocytes) occurs through both active and passive diffusions (Crespy et al., 2003; Vaidyanathan and Walle, 2003; Lambert et al., 2007; Neilson and Ferruzzi, 2012). Polyphenols appear to compete for the monocarboxylic acid transporter (Bravo, 2009). Passive diffusion appears to contribute to the absorption of flavonoids with high log *P* values such as isoflavones and flavonones but it contributes little to those with a low log *P* value such as flavan3-ols (Neilson and Ferruzzi, 2012).

#### Flavonoids

Flavonoids are the largest class of plant polyphenols present in fruits and vegetables. There are more than 4,000 distinct flavonoids identified to date (Shahidi and Naczk, 1995). The main subclasses of flavonoids common in diet are flavones (e.g., luteolin and apigenin), flavonols (e.g., quercetin, kaempferol, and myricetin), flavon-3-ols (catechin, epicatchin, epigallocatechin, epicatechin gallate, and apigallocatechin gallate), isoflavones (e.g., genistein and daidzein), flavonones (e.g., naringenin), and anthocyanidins (e.g., cyaniding and malvidin; Liu, 2012). This class of polyphenols has received attention due to their potent anti-oxidant activity (Rice-Evans et al., 1995) and possible role in the prevention of cancer (Birt et al., 2001), cardiovascular (Hooper et al., 2008), neurodegenerative (Nakajima et al., 2007), and infectious diseases (Shin et al., 2005).

Flavonoids are often recognized as xenobiotics by the intestinal detoxification system (Jeong et al., 2005). They are oxidized by phase I enzymes, conjugated by phase II enzymes and then excreted from the cells by phase III transporters (Shimizu, 2010). Recent studies have observed that the detoxification enzymes are regulated by a variety of transcription factors and regulatory proteins (Kusano et al., 2008).

With the exception of catechins (which have a notable presence in tea and are also found in fruits), flavonoids in nature are almost always found as a glycoside, i.e., attached to a sugar group (Aherne and O’Brien, 2002). The aglycone, which is the flavonoid without the sugar, is not normally found in food; however, the processing of plant food such as fermentation can increase the level of aglycone such as in the case of miso soup. Glycosylation increases the polarity of flavonoids which is important for storage in the plant cell vacuoles. Flavonols and flavones occur in food usually as o-b-glycosides (Aherne and O’Brien, 2002). Of the major flavonoid classes, the flavonols predominate in fruits in which a variety of glycosides have been identified, whereas in vegetables quercetin glycosides predominate (Aherne and O’Brien, 2002). When glycosides are formed, the preferred glycosylation site on the flavonol molecule is the C-3 position and less frequently the C-1 position (Aherne and O’Brien, 2002). D-glucose is the most usual sugar residue but other carbohydrate substitutions include arabinose, galactose, glucorhamnose, lignin, I-rhamnose, and xylose 4 (Aherne and O’Brien, 2002). For example, quercetin can be linked to the 3-o-glycoside rhamnose to yield quercitrin, or glucorhamnose to yield rutin (Aherne and O’Brien, 2002). Flavonols are found in nearly all fruits and vegetables with quercetin glycosides being the most abundant in the diet (Psahoulia et al., 2007).

#### Polyphenols in health and disease

Cross-sectional and prospective epidemiologic studies have found an association with diets rich in plant foods and protection against degenerative diseases such as cancer and cardiovascular diseases (CVDs; Hertog et al., 1993b; Omenn et al., 1996; Liu et al., 2005; Scalbert et al., 2005; Kuriyama et al., 2006; Del Rio et al., 2010; Sies, 2010). Intervention studies in humans and animals have contributed further evidence supporting polyphenolic modulation of vascular and platelet function, blood pressure (Hodgson and Croft, 2006), and improved plasma lipid profile (Scalbert et al., 2005). A review analyzed 200 studies on the relationship between intake of fruits and vegetables and different types of cancers (Block et al., 1992). In 128 of the 156 dietary studies, the consumption of fruit and vegetables was reported to be significantly protective. The risk of cancer was twofold higher in those individuals that had relatively low fruit and vegetable intake (Block et al., 1992). Several epidemiological studies have examined the role of phytochemicals on CVD prevention. Dietary flavonoid intake was significantly linked to reduced heart disease in general as well as coronary heart disease specifically related mortality (Hertog et al., 1993a, 1995).

There have been several *in vivo* studies with polyphenols (reviewed by Gonzalez et al., 2011). These studies have indicated that polyphenols help in the regulation of diseases including immunoregulation, estrogen modulation, and protease inhibition in rheumatoid arthritis; immunoregulation in experimental allergic encephalomyelitis (a model for multiple sclerosis); anti-inflammatory effects in inflammatory bowel disease; anti-allergic effects in asthma; anti-inflammatory and anti-oxidant effect, transcription factor regulation, and protective mechanisms in atherosclerosis; anti-inflammatory and protection against tissue damage in ischemia-reperfusion; anti-inflammatory and anti-oxidant effects and control of hyperinsulinemia, hypertension, dyslipidemia in metabolic syndrome, and skin inflammation (Gonzalez et al., 2011).

There have been several *in vitro* studies related to the anti-inflammatory, anti-oxidant, and immunomodulatory actions of polyphenol and in particular flavonoids (reviewed by Gonzalez et al., 2011). The phytochemical chlorogenic acid (found in many agricultural products such as coffee and apples) and its metabolite caffeic acid have been shown to reduce the secretion of the proinflammatory cytokine IL8 in the human IECs caco2, when they were stimulated with TNFα and H_2_O_2_ (Zhao et al., 2008). Similar results were found for isoflavone fractions in another study (Satsu et al., 2009). Chlorogenic acid was also observed to inhibit LPS induced cyclooxygenase-2 expression in mouse macrophage cells, by suppressing NFκβ activation (Shan et al., 2009).

*In vitro* studies have also shown that dietary substances including polyphenols, can be modulators of tight junctions in the intestinal epithelium (Suzuki et al., 2011; Kosiñska and Andlauer, 2013). In addition, polyphenols have been reported to modulate transporter function. A study reported that glucose absorption via the intestinal SGLT1 was slightly inhibited in rats by hydrolyzed metabolites from gymnemic acid extracted from *Gymnema sylvestre* leaves (Yoshikawa et al., 1997). Another study looked at the effect of polyphenols from Cocoa on T84 colonic epithelia in Ussing chambers on the forskolin-stimulated cystic fibrosis transmembrane conductance regulator (CFTR; Schuier et al., 2005). CFTR is the major chloride ion channel in the apical membrane of the epithelia and it is critically involved in salt and water secretion and absorption in the gastrointestinal tract and other epithelial membranes. Pharmacologic blocking of CFTR is thought to inhibit salt and water loss during diarrhea. They found that cocoa flavonols act as a mild CFTR blocker. The authors noted that flavonols are poorly absorbed in the small intestine and therefore large amounts of the compound would be present in the intestinal lumen to interact with the apical surface of the IECs (Schuier et al., 2005).

*In vitro* studies have also highlighted that phytochemicals may have detoxification properties. pregnane X receptor (PXR) is involved in the recognition of xenobiotics and upregulation of the detoxification enzymes which help in metabolizing harmful compounds and excreting them (Shimizu, 2010). A study looked at the effect of food substances on PXR-mediated regulation of the detoxification enzymes using human intestinal LS180 cells (Satsu et al., 2008). Of the 42 phytochemicals tested, three flavonoids and two terpenoids activated PXR-dependent transcriptional activity suggesting that these compounds activate the intestinal detoxification system and are involved in the barrier function against toxic chemicals (Satsu et al., 2008; Shimizu, 2010). In addition, food substances have also been shown to bind toxin directly, interfering with their absorption through the intestine (Natsume et al., 2005). These studies suggest that phytochemicals are not only processed by the epithelium but also influence and modulate it.

One study has investigated the anti-oxidant capacity of intact juice blend in both *in vivo* and *in vitro* models (Jensen et al., 2008). The authors initially established that their juice blend to contained major polyphenol compounds including anthocyanins, proanthocyanidins, and phenolic acids. Using a CAP-e assay they established that their juice blend is able to provide anti-oxidant protection *in vitro*. They also found that ROS productions were reduced in polymorphonuclear leukocytes cells *in vitro* after incubation with the juice blend. They then went on to testing the juice blend *in vivo* using a randomized, placebo-controlled trial using 12 individuals in a within subject design. Using the CAP-e assay they observed that there is an increase in anti-oxidant capacity within 1 and 2 h of consuming the juice blend (Jensen et al., 2008). In 2011, a pilot study was performed to evaluate the effect of the juice blend on individuals with reduced range of motion (ROM) due to pain (Jensen et al., 2011). The study suggested that the juice blend increased anti-oxidant levels in serum (using the CAP-e assay) and this was correlated with improved ROM and reduced pain. The authors state that while the results look promising, the significant association among increased anti-oxidant status, improved ROM, and pain reduction warrants further study (Jensen et al., 2011). Even if *in vitro* simulators suffer from the absence of a complete physiological environment, they are still valuable to study the intestinal processes in the gut itself without ethical constraints (Bolca et al., 2013).

#### Whole food, native compounds, and synergistic effect

It is important to note that the observed health benefit of phytochemicals may not necessarily occur due to the native form that is found in food (Neilson and Ferruzzi, 2012). This is because of the various metabolic processes that occur after absorption. These metabolic processes that are performed by the digestive enzymes and the gut microflora, breakdown the phytochemicals into simpler compounds and alter the functional groups of the phytochemical. Therefore the metabolites may actually be the active compound responsible for the biological activity. However, many studies measure the biological activity of the native phytochemical for several reasons: (1) most phytochemicals can be converted into many metabolites which exponentially increases the number of metabolites that need to be measured, (2) in many situations, the metabolites that are generated from a phytochemical are unknown or incomplete, (3) the activity of the native phytochemical is better characterized than its metabolites both *in vivo* and *in vitro*, and (4) the native compound serves as a marker for all its metabolites even if not a complete one (Neilson and Ferruzzi, 2012).

It is believed that the observed beneficial activities of phytochemicals from fruit and vegetables are more likely due to a combined effect rather than to a single compound or small group of compounds (Neilson and Ferruzzi, 2012). This is because when looked at in isolation the individual phytochemical studied in clinical trials do not appear to have consistent preventative effects (Omenn et al., 1996; Stephens et al., 1996; Yusuf et al., 2000). The isolated compound either loses its bioactivity or may not behave the same way compared to when it is in whole foods. Several studies have shown that the risk of cancer is inversely linked to eating green and yellow vegetables and fruit. B-carotene, which is present in abundance in these fruits and vegetables, was therefore extensively studied as a possible cancer-preventative agent. However, the result from several clinical studies were inconsistent (Greenberg et al., 1990; Hennekens et al., 1996; Omenn et al., 1996). In one study, the incidence of skin cancer was unchanged in patients receiving a b-carotene supplement (Hennekens et al., 1996). In the Heart Outcomes Prevention Evaluation (HOPE) study, patients at a high risk for CVD were given vitamin E supplement or placebo (Jialal et al., 2000). No difference was found in CVD mortality (Jialal et al., 2000).

Other studies have also reported on the negative impact of anti-oxidant supplements. A systemic review in 2012 assessed the effect of anti-oxidant supplements on mortality and health compared to placebo or no intervention (Bjelakovic et al., 2012). They analyzed 78 trials and concluded that there was no evidence of benefit from consuming anti-oxidant supplements. Moreover, they found that consumption of b-carotene, vitamin E and high concentrations of vitamin A may be harmful and increase risk of mortality (Bjelakovic et al., 2012). In another study, the association between lung cancer and b-carotene was investigated in smokers (Omenn et al., 1996). Smokers gained no benefit from the supplement and the authors suggested that there may in fact have been a significant increase in lung cancer and mortality (Omenn et al., 1996).

There are thousands of phytochemicals present in whole foods which differ in their molecular size, polarity, and solubility (Liu, 2012). These properties may affect their bioavailability and distribution on different macromolecules, subcellular organelles, cells, organs, and tissues (Liu, 2012). It is thus more likely that phytochemicals work synergistically to produce their therapeutic effect. A synergistic therapeutic effect is defined as a stronger effect by the combination of two or more compounds compared to individual compounds at equal concentrations (Yang and Liu, 2009).

Evidence for a synergistic therapeutic effect was seen in apple studies. A study looked at the effect of phytochemicals extracted from whole apple on tumor cell growth *in vitro* (Eberhardt et al., 2000). They found that whole apple extracts inhibited colon cancer proliferation in a dose-dependent manner with extracts equivalent to 0–50 mg/ml of whole apple wet weight. The phytochemicals in apples with peel exhibited a stronger effect compared to apple without peel (Eberhardt et al., 2000). Another study built on this information by performing an animal study (Liu et al., 2005). They demonstrated that apple extracts prevented mammary cancer in rats in a dose-dependent manner comparable to human consumption of 1, 3, and 6 apples a day (Liu et al., 2005). More recently, a study examined the potential additive, synergistic or antagonistic interaction among apple phytochemicals (Yang and Liu, 2009). The results suggested that apple phytochemicals in combination with quercetin 3-beta-D-glucoside (Q3G) possesses a synergistic effect against MCF-7 human breast cancer cell proliferation (Yang and Liu, 2009).

## NUTRIENT MODULATION IN AUTOPHAGY AND BACTERIAL SENSING

It is emerging that nutrients have the ability to modify various cellular processes in particular autophagy (Marion-Letellier et al., 2013). Inducing autophagy through the administration of different nutrients may be beneficial for intestinal inflammation.

Many recent studies have reported the interaction between autophagy and dietary factors. This includes the amino acids arginine (arg), glutamine (gln), and leucine (leu) which play a crucial role in intestinal growth, integrity, and function through cellular mechanisms (Rhoads and Wu, 2009). It is becoming clear that mTOR signaling plays a part in modulating amino acid intestinal homeostasis (Goberdhan et al., 2009). Studies have reported that arg, gln, and leu regulate the mTOR pathway (Goberdhan et al., 2009). Arg has been shown to upregulate phosphorylation of S6K, a downstream effector of mTOR (Ban et al., 2004). Gln induces autophagy through the mTOR and p38 MAPK pathways (Sakiyama et al., 2009).

Flavonoids from diet, such as dihydrocapsaicin (DHC), quercetin, MK615, and soyasaponins, induce autophagy in the intestine; however, the mechanism of action is still undetermined (Marion-Letellier et al., 2013). The polyphenol quercetin was reported to induce autophagy in Caco-H2 intestinal cell line with oncogenic Ras activity that resulted in preferential reduction of the Ras protein (Psahoulia et al., 2007). Saponins derived from soy bean were shown to suppress HCT15 colon cancer cell proliferation through S-phase cell-cycle delay, and can induce macroautophagy suggesting autophagic cell death (Ellington et al., 2005). Incubation with an extract from Japanese apricot, MK615 resulted in an induction of autophagy in the colon cancer cell line (Mori et al., 2007).

Peroxisome proliferator-activated receptor gamma (PPARγ) which is important in the regulation of inflammation is also thought to regulate autophagy (Jiang et al., 2010). Polyunsaturated fatty acids (PUFAs) and resveratrol have been shown to induce PPARγ which is highly active in the colon (Marion-Letellier et al., 2009). It has been shown that fatty acids such as docosahexaenoic acid (*DHA*) may be potent inducers of autophagy through PPARγ in intestinal cells (Marion-Letellier et al., 2009).

A study reported that the stimulation of autophagy by treatment with vitamin D significantly enhanced the anti-microbial response against *M. tuberculosis* in human macrophages. This effect seemed to be dependent on cathelicidin, a peptide that is activated by vitamin D and enhances co-localization of bacterial phagosomes with autophagosomes (Yuk et al., 2009).

In the Department of Nutrition at the University of Auckland, our 6-week intervention study assessed the effect of a Mediterranean diet on inflammation (Ellett et al., 2013). During the study, blood samples were taken at the beginning and the end of the trial. CRP levels were measured as a marker of inflammation and gene expression was measured using gene arrays. At the end of the 6 weeks, CRP levels decreased and a significant change in gene expression was observed. The change in gene expression included TLR4 and TLR2 indicating that the TLR pathway is modulated by changes in diet.

It is possible that chemical antagonists of NOD1 and NOD2 could have a therapeutic application for diseases where dampening the inflammatory response would be beneficial (Correa et al., 2012). A study has reported that certain arene-Cr(CO)3 complexes decrease inflammatory responses and reduce NOD2-mediated inflammatory pathways (Bielig et al., 2010). These compounds seem to be specific for NOD2 but not TLRs or TNFα receptors (Bielig et al., 2010).

The studies highlighted here only provide a mere glimpse on the potential of using dietary intervention to modify and prime different physiological processes including immunity. The interaction between diet and the internal environment is not a new concept. However, understanding and manipulating this interaction at a molecular level to gain conclusive benefit remains an ambitious task due to the interdisciplinary nature of this subject. The bacterial sensing machinery was the focus of this review as it offers a good biological process to study as they are important in sensing and responding to the external environment. Nonetheless there are other pathways that may be more relevant to a particular disease or condition and would also be worth studying. It is likely that understanding these pathways further and how diet can interact with them will contribute to developing personalized nutrition to manage disease.

## CONCLUSION

In this review, we introduced some of the main PRRs and autophagy and how they function. Additionally we reviewed dietary phytochemicals which are believed to be associated with health and wellbeing. Dietary interactions with the host biological processes for therapeutic purposes have been the subject of great interest and thousands of studies and clinical trials. This review was an attempt to lay down the foundations of what is already known from literature in order to help develop personalized nutrition further for better management of disease.

## Conflict of Interest Statement

The authors declare that the research was conducted in the absence of any commercial or financial relationships that could be construed as a potential conflict of interest.
